# Expression of *KCNQ1OT1*, *CDKN1C*, *H19*, and *PLAGL1* and the methylation patterns at the KvDMR1 and *H19/IGF2* imprinting control regions is conserved between human and bovine

**DOI:** 10.1186/1423-0127-19-95

**Published:** 2012-11-15

**Authors:** Katherine Marie Robbins, Zhiyuan Chen, Kevin Dale Wells, Rocío Melissa Rivera

**Affiliations:** 1 Division of Animal Sciences, University of Missouri, Columbia, MO, USA

**Keywords:** KvDMR1, H19/IGF2 ICR, KCNQ1OT1, CDKN1C, PLAGL1, Beckwith-Wiedemann syndrome, Methylation, Genomic imprinting, Epigenetics, Bovine

## Abstract

**Background:**

Beckwith-Wiedemann syndrome (BWS) is a loss-of-imprinting pediatric overgrowth syndrome. The primary features of BWS include macrosomia, macroglossia, and abdominal wall defects. Secondary features that are frequently observed in BWS patients are hypoglycemia, nevus flammeus, polyhydramnios, visceromegaly, hemihyperplasia, cardiac malformations, and difficulty breathing. BWS is speculated to occur primarily as the result of the misregulation of imprinted genes associated with two clusters on chromosome 11p15.5, namely the KvDMR1 and *H19/IGF2.* A similar overgrowth phenotype is observed in bovine and ovine as a result of embryo culture. In ruminants this syndrome is known as large offspring syndrome (LOS). The phenotypes associated with LOS are increased birth weight, visceromegaly, skeletal defects, hypoglycemia, polyhydramnios, and breathing difficulties. Even though phenotypic similarities exist between the two syndromes, whether the two syndromes are epigenetically similar is unknown. In this study we use control *Bos taurus indicus* X *Bos taurus taurus* F1 hybrid bovine concepti to characterize baseline imprinted gene expression and DNA methylation status of imprinted domains known to be misregulated in BWS. This work is intended to be the first step in a series of experiments aimed at determining if LOS will serve as an appropriate animal model to study BWS.

**Results:**

The use of F1 *B. t. indicus* x *B. t. taurus tissues* provided us with a tool to unequivocally determine imprinted status of the regions of interest in our study. We found that imprinting is conserved between the bovine and human in imprinted genes known to be associated with BWS. *KCNQ1OT1* and *PLAGL1* were paternally-expressed while *CDKN1C* and *H19* were maternally-expressed in *B. t. indicus* x *B. t. taurus* F1 concepti. We also show that in bovids, differential methylation exists at the KvDMR1 and *H19/IGF2* ICRs.

**Conclusions:**

Based on these findings we conclude that the imprinted gene expression of *KCNQ1OT1*, *CDKN1C*, *H19*, and *PLAGL1* and the methylation patterns at the KvDMR1 and *H19/IGF2* ICRs are conserved between human and bovine. Future work will determine if LOS is associated with misregulation at these imprinted loci, similarly to what has been observed for BWS.

## Background

Genomic imprinting is an epigenetic modification that directs parent-specific gene expression. Imprinted genes are responsible for regulating growth and development of the conceptus [1]. These genes are typically found in clusters containing both maternally- and paternally-expressed genes. The correct allelic expression of the clustered genes is regulated by a neighboring region of DNA which is differentially methylated and is known as the imprinting control region (ICR; [2-4]). The effect of the ICR on a cluster of imprinted genes can span for megabases in a bidirectional manner [5].

Imprinted genes are functionally haploid [6] and therefore are vulnerable to epigenetic mutations and loss-of-imprinting (LOI; [7]). LOI refers to the misregulation of imprinted gene expression which results in either loss of expression or biallelic expression of these genes.

There are several LOI disorders in humans including Beckwith-Wiedemann syndrome (BWS), Angelman syndrome, Prader-Willi syndrome, and Silver Russell syndrome. BWS is the most frequent LOI syndrome observed in humans with an incidence of one in 13,700 live births [8,9]. BWS is also the most common pediatric overgrowth syndrome [9]. The overgrowth parameters for height and weight for BWS patients are among the 97^th^ percentile [9].

The primary features of BWS include macroglossia, macrosomia, and abdominal wall defects [10,11]. The secondary features include visceromegaly, polyhydramnios, renal abnormalities, facial nevus flammeus, hypoglycemia, hemihyperplasia, ear creases and helical pits, and cardiac malformations [9-12]. Children with this syndrome also have an increased susceptibility (4–21%) to develop embryonic tumors by the time they turn five years of age [8,13,14]. Wilms’ tumor of the kidney is the most common embryonic tumor (67% of cases) observed in BWS patients [14].

BWS is thought to occur because of the dysregulation of several imprinted genes located primarily on chromosome 11p15.5 [9,11,15]. The two main imprinted gene clusters associated with BWS are those directed by the KvDMR1 and *H19/IGF2* ICRs [12,16]. The BWS-associated imprinted genes regulated by the KvDMR1 include the paternally-expressed non-coding RNA *KCNQ1OT1* and the maternally expressed coding genes *CDKN1C*, *KCNQ1*, and *PHLDA2*. In mice, expression of *CDKN1C* is also regulated by a differentially-methylated region (DMR) of DNA that encompasses the promoter and extends through exon 2 [17,18]. Contrary to what has been reported for mice, no differential methylation is observed for *CDKN1C* in humans [19].

The KvDMR1 is methylated on the maternal allele and unmethylated on the paternal allele in mouse and human. Loss of methylation (LOM) at the KvDMR1 on the maternal allele is the most common epigenetic defect (50%) observed in BWS patients [9,12,16,20,21]. This LOM results in the aberrant expression of the long noncoding RNA (ncRNA) *KCNQ1OT1* from the maternal allele which results in bidirectional silencing of the maternally-expressed flanking genes, in particular *CDKN1C*[8,22].

The *H19/IGF2* ICR regulates the expression of the paternally-expressed gene *IGF2* and the maternally-expressed ncRNA *H19*. This region is unmethylated on the maternal allele and methylated on the paternal allele [12]. The gain of methylation on the maternal allele results in the repression of *H19* from the maternal allele leading to biallelic expression of *IGF2*. This epimutation occurs in 2–10% of BWS patients and is highly associated with tumor development [9,16,23]. Recent studies have found that some BWS patients also have LOM at the *HYMAI/PLAGL1*, *MEST*, and *GRB10* ICRs [24-26].

In humans *PLAGL1* is found on chromosome six, unlike the other genes associated with BWS which are found primarily on chromosome 11. *PLAGL1* functions as a tumor suppressor and can induce apoptosis [27,28]. In a study by Arima *et al.,*[27] it was determined that *PLAGL1* is expressed similarly to *CDKN1C* in many tissues. A recent microarray study [29] places PLAGL1 as a pivotal player in the regulation of expression of a network of imprinted genes, including *H19*, *IGF2*, and *CDKN1C*.

In ruminants there is an overgrowth syndrome that resembles BWS. The overgrowth syndrome in ruminants is known as large offspring syndrome (LOS; [30]). LOS has been documented to result from several embryo culture conditions [31-34] and high protein diet supplementation to the dam prior to conception and during early pregnancy [35]. The phenotypical features of LOS include: increased birth weight, macrosomia, skeletal defects, hypoglycemia, polyhydramnios, visceromegaly, difficulty suckling, and perinatal death [30,31,36-38].

Currently, no animal models exist that recapitulate the overgrowth phenotype of BWS. Murine knockout models for BWS have been unable to display all the primary features observed in children with BWS [39]. As an effort to develop treatments for BWS symptoms, our long-term goal is to determine if LOS in ruminants can be used as an animal model to understand the etiology of the LOI syndrome BWS. The goal of this paper was to ascertain baseline allelic expression and DNA methylation in control bovine concepti of imprinted genes/regions known to be misregulated in BWS. Similar to what has been previously reported [40,41]; we show that *KCNQ1OT1*, *H19*, *CDKN1C* and *PLAGL1* are imprinted in the bovine. In addition, we confirm that the KvDMR1 and *H19/IGF2* ICR are differentially methylated in the bovine genome which is in accordance to what has been reported in humans. Our study extends previous work [40,41] in that it provides fixed DNA sequence polymorphisms between *Bos taurus indicus* and *Bos taurus taurus* that can be used to distinguish with certainty the parental alleles in F1 individuals.

## Methods

### DNA sequence polymorphism identification

The ability to differentiate between parental alleles in an F1 individual is fundamental when performing genomic imprinting studies. For our studies we used two subspecies of cattle (*Bos taurus taurus, Bos taurus indicus),* which diverged ~620,000 years ago [42], to produce F1 individuals. Studies have shown that single nucleotide polymorphisms (SNP) should be found every 172 base pairs (bp) within the exon regions of genes between *B. t. taurus* and *B. t. indicus*[43,44]. Genomic regions sequenced included the exons of *KCNQ1OT1, H19, CDKN1C,* and *PLAGL1* as well as the KvDMR1 and *H19/IGF2* ICRs. Table 1 shows the subspecies-specific single nucleotide polymorphisms (SNPs) for these regions.

**Table 1:** DNA sequence polymorphisms used to ascertain allele-specific expression and methylation

**Gene/ICR Symbol**	**Type of assay**	**Maternal (B.t. taurus)**	**Paternal (B.t. indicus)**	**Location of primers within the gene**	**NCBI accession # (based on Btau-4.2)**	**PM location in reference Btau 4.2**
H19	gene expression	C	T	Exon 2–5	NR_003958.2	1831
KCNQ1OT1	gene expression	A	G	Exon 1 (close to the start of transaction)	NW_001494547.3	3146321
CDKN1C	gene expression	C	T	Exon 2–4	NW_001494547.3	2955801
PLAGL1	gene expression	T	G	Exon 6	NM_001103289.1	867
H19/IGF ICR	DNA methylation	A	G	N/A	NW_001494547.3	3724402
KvDMR1	DNA methylation	G, A, A in/del between GA, C^**^	C^**^, G, G, in/del between GA “GCG”, G	N/A	NW_001494547.3	3134118, 3134110, 3134095, 3134087-3134086, 3134072
CDKN1C DMR	DNA methylation	N/A	N/A	Exon 1-Intron 2	NW_001494547.3	N/A

PM=polymorphisms, N/A=not applicable, C**= During bisulfite conversion the C is converted to a T.

### Production of *Bos taurus indicus x B. taurus taurus* day 65 F1 concepti

**Figure 1:**
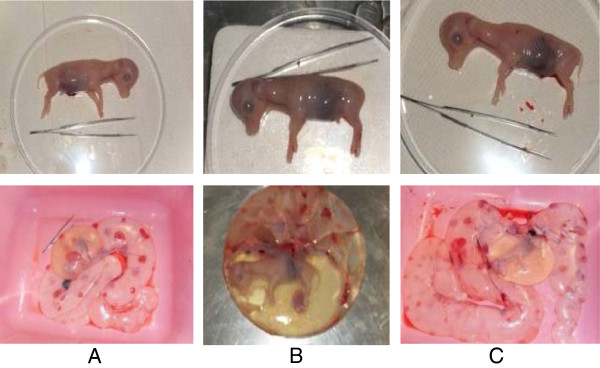
**Day 65 *B. t. indicus *x *B. t. taurus *F1 concepti.** The tissues from these concepti were used to determine baseline imprinted gene expression and DNA methylation in bovine of BWS-associated loci.

All animal work was done in accordance with the University of Missouri Animal Care and Use Committee. The estrous cycles of seven *B. t. taurus* heifers (6 Angus, 1 Hereford) were synchronized using the 14-CIDR®-PG (Controlled Intravaginal Drug-Releasing Device and Prostaglandin*)* estrus synchronization protocol. Briefly, CIDRs were inserted for 14 days to suppress progesterone levels. Sixteen days after the removal of the CIDRs, 25 mg of prostaglandin F_2_ alpha (Lutalyse; dinoprost tromethamine; Pfizer Animal Health, New York, NY) was administered intramuscularly (i.m.). Three days after prostaglandin injection, 100 mcg of gonadotropin releasing hormone was administered i.m. (Cystorelin; gonadorelin diacetate tetrahydrate; Merial; Duluth, GA). Heifers were then artificially inseminated with semen from one *B. t. indicus* bull (Nelore breed; ABS CSS MR N OB 425/1 677344 29NE0001 97155). Three out of the seven heifers (2 Angus, 1 Hereford) were confirmed pregnant by ultrasonographic examination on day 30 of gestation. Two males and one female *B. t. indicus* x *B. t. taurus* F1 concepti were collected on day 65 of gestation at the University of Missouri Veterinary School’s abattoir (Figure 1).

Concepti were collected on day 65 because a study by Cezar *et al.*[45] determined that DNA methylation levels were the same between a day 60 fetus and an adult animal. The following tissues were collected: amnion, chorioallantois, brain, tongue, heart, kidney, liver, lung, intestines, and reproductive tract. Tissues were snap frozen in liquid nitrogen and stored at −80°C until use.

### RNA extraction and cDNA synthesis for parental-allelic expression analysis

The chorioallantois, liver, brain, heart, and tongue of day 65 *B. t. indicus x B. t. taurus* F1 concepti were homogenized with a plastic disposable pestle (Fischer Scientific; Pittsburgh, PA) in 450μl of lysis binding buffer (4.5M guanidine-HCl, 50mM Tris-HCl, 30% Triton X-100 (w/v), pH 6.6). The tissue lysates were then passed through a 22 and 26 gauge needles connected to a 1ml syringe. RNA was extracted from the tissues using a commercially available kit (High Pure RNA; Roche Applied Science; Mannheim, Germany) following manufacturer’s specifications.

cDNA was synthesized in a 20μl reaction using 10μl of RNA (130 ng Total RNA) and 10μl of a master mix containing: 10 mM DTT (Invitrogen; Carlsbad, CA), 1X First Strand buffer (Invitrogen; Carlsbad, CA), 0.5 μg random primers (Promega; Madison, WI), 1mM dNTPs (Fischer Scientific; Pittsburgh, PA), 100 units Superscript II reverse transcriptase (RT; Invitrogen; Carlsbad, CA), and 20 units of Optizyme RNase Inhibitor (Fischer Scientific; Pittsburgh, PA). The samples were then incubated in a thermal cycler for one hour at 42°C followed by ten minutes at 95°C. The samples were then stored in the −20°C until further analysis. To verify the absence of DNA contamination, a control was prepared for each sample without Reverse Transcriptase. RNA was also collected and cDNA prepared from several *B. t. taurus* and *B. t. indicus* tissues to serve as restriction fragment length polymorphism (RFLP) assay controls.

### Imprinted expression analysis of *B. t. indicus* x *B. t. taurus* concepti

*B. t. indicus* x *B. t. taurus* F1 tissues were used to determine gene expression of *KCNQ1OT1*, *CDKN1C*, *H19*, and *PLAGL1*. The PCR primers generated for expression analyses were intron-spanning for *CDKN1C* and *H19*. However, the primers used to amplify *KCNQ1OT1* and *PLAGL1* were designed within a single exon. The possibility of DNA contamination in the cDNA was assessed by the exclusion of the Reverse Transcriptase from the cDNA master mix in parallel samples. The conditions used for RT-PCR were modified until a single amplicon was observed for each primer set. The RT-PCR program started with an initial denaturation step at 94°C for 2:15 min. The denaturation (94°C for 30 sec), annealing (refer to Table 2), and extension (72°C for 1 min) steps were repeated for the specified cycle number on Table 2. The PCR programs ended with a five minute extension at 72°C. The identity of PCR products was confirmed by restriction enzyme digest or sequencing. No further optimization for sensitivity was required. Primer and PCR condition information may be found in Table 2.

**Table 2:** PCR primers and conditions used determine imprinted gene expression and DNA methylation

**Gene/ICR Symbol**	**Primers (5′-3′)**		**PCR Annealiang Tm (°C)**	**PCR size (bp)**	**Primer [] μM**	**MgCI**_**2**_**(9mM)**	**#Cycles**
H19	Forward	GATATGGTCCGGTGTGATGGAGAGAGCA	62.8	752	0.3	2.5	35
	Reverse	TTCGGAGCCTCCAGACTGCGGTG					
KCNQ1OT1	Forward	TCGAGGGTACCGGATTCCCAGGC	64	502	0.3	2.5	35
	Reverse	CGCAGGACACCCCAACTACAGCC					
CDKN1C	Forward	GGAGGCGCCGCGATCAAGAAG	62	745	0.3	4	35
	Reverse	GACAGCGAAAGCGCGAAGAGAC					
PLAGL1	Forward	TCAACCGGAAAGACCACCTGAAGA	60	834	0.3	4	35
	Reverse	GGTCAAAGCCTGCATTGAGCTTGT					
H19/IGF2ICR	Forward	GGGGAGGTTGTCGGGTTTATGG	60	493	0.3	2.5	40
	Reverse	CCGCACCCCTCCTTTAACATC					
KvDMR1	Forward	TGAGGAGTGAGTTATGAGGA (taurus) TGAGGATTGTAGTTGTGAGGA (indicus)	59.2	419/422	0.3	4	45
	Reverse	CTACCACATCTACCCCAATC					
CDKN1C DMR	Forward	GAGGACTGGGCGTTCCACAGGCCA	62	1108	0.4	GC Buffer II Takara	35
	Reverse	GCCCTTTAACGGCCAGGAGGC					

Tm= Temperature in °C, bp= base pairs, [] = concentration.

RFLP was used to identify allelic expression for each gene. The SNPs responsible for restriction site polymorphisms between *B. t. taurus* and *B. t. indicus* are shown in Table 2. After restriction enzyme digestion the assays were resolved by polyacrylamide gel electrophoresis (PAGE; Table 3). For cases in which the repressed allele was expressed the band intensity was measured by the UN-SCAN-IT gel 5.3 alias gel analysis software (Silk Scientific; Orem, UT) that functions as a gel band densitometer. To be considered biallelic a sample had to have 10% or higher expression from each parental allele [46].

**Table 3:** Restriction enzymes used to determine allele-specific expression of imprinted genes

**Gene Symbol**	**Expressed Allele**	**Restriction enzyme**	**Digested B t. taurus (bp)**	**Digested B. t. indicus (bp)**	**PAGE Details**
H19	Maternal	BsiHKAI	609,143	609,35,108	18%
KCNQ1OT1	Paternal	Hinfl	457,32,13	268,189,32,13	7%
CDKN1C	Maternal	Avall	494,251	361,251,133	10%
PLAGL1	Paternal	Mlul	834	387,447	10%

PAGE= Polyacrylamide gel eclectrophoresis, bp= base pairs.

### DNA extraction, bisulfite mutagenesis and COBRA procedures

DNA was extracted from day 65 *B. t. indicus x B. t. taurus* F1 tissues using a phenol-chloroform extraction procedure. Bisulfite mutagenesis was then performed following the instructions for the Imprint DNA Modification Kit One-Step procedure (Sigma-Aldrich; St. Louis, MO). During the bisulfite mutagenesis procedure all unmethylated cytosines are converted to uracils while methylated cytosines remain cytosines. During PCR the uracils are replaced by thymines. Primers for the bisulfite mutagenized DNA were designed for the *H19/IGF2* ICR and the KvDMR1 (Table 2)*.* PCR was used to amplify a 493 bp region of the *H19/IGF2* ICR. The amplicon size for the KvDMR1 was 419 bp for the *taurus* allele and 422 bp for the *indicus* allele as a result of an insertion/deletion in the DNA sequence. For the KvDMR1, allele-specific bisulfite primers were designed to amplify each parental allele. The rationale for this was based on the location of the fixed polymorphic sites between the two subspecies of cattle as identified by Sanger sequencing. In order to use the polymorphisms to determine parental-specific methylation primers were required within a region that is 1936 bp, 67% GC, flanked by repeat sequences and contains additional polymorphisms. No single primer set was identified that amplified both alleles. Manual design of allele-specific primers allowed for amplification of each KvDMR1 allele separately but in the same reaction. After bisulfite mutagenesis, amplicons from differentially methylated alleles can be recognized by RFLP.

Methylation status of the loci was first determined by combined bisulfite restriction enzyme assay (COBRA). This assay was also used to ascertain that both the methylated and the unmethylated alleles amplified equally with no amplification biased was introduced during PCR. The enzymes used to digest the originally methylated alleles were DpnII and BstUI for the *H19/IGF2* ICR and the KvDMR1, respectively. The PCR amplicons and digested products were resolved by 7% PAGE.

### DNA Methylation analysis of the KvDMR1 and *H19/IGF2* ICR

Bisulfite-converted DNA amplicons were isolated from agarose gels using the Wizard SV gel and PCR Clean-Up System (Promega, Madison, WI). *H19/IGF2 ICR* amplicon was cloned using the pGEM T Easy Vector System ligation buffer protocol (Promega). The plasmid was transformed into chemically competent NEB 5-alpha F’I^q^*E.Coli* cells (New England BioLabs; Ipswich, MA) according to the manufacturer’s instructions. The KvDMR1 amplicon was cloned using CopyControl PCR cloning kit with TransforMax™ EPI300™ Electrocompetent *E. coli* cells (Epicenter Biotechnologies) according to the manufacturer’s specifications except that all the incubation procedures were done at room temperature. Next, the individual clones were sequenced at the University of Missouri’s DNA Core using the 96-capillary Applied Biosystems 3730 DNA Analyzer with Big Dye Terminator.

### Determination of the methylation status of *CDKN1C* in bovine

In the mouse, *CDKN1C’s* DMR has been shown to extend from the promoter region through the second exon. However, the homologous region is not differentially methylated in humans. Many attempts (>30 primer pairs were tested) were made to amplify the promoter of the *CDKN1C* gene in bovine [NW_001494547.3; 2951474-2953864]. However, sequencing results never coincided with the expected region on chromosome 29 although, according to the databases, the primers aligned perfectly to the bovine *CDKN1C’s* promoter. In addition, even though we were able to sequence *CDKN1C*’s exons one and two and intron one, those regions lacked SNPs between *B. t. taurus* and *B. t. indicus*. Therefore, we undertook a PCR based methylation analysis to determine if the putative bovine DMR was methylated as in mice or unmethylated as in humans.

Isoschizomers were used to test the methylation status of *CDKN1C* (HpaII and MspI). These two restriction enzymes allowed differentiation between methylated and unmethylated CpGs. HpaII is methylation sensitive and blocked by CpG methylation and therefore is not be able to cut genomic DNA that is methylated at the CCGG recognition sites. However, MspI is a methylation insensitive restriction enzyme and is able to cleave both methylated and unmethylated DNA at the CCGG recognition sites.

First, genomic DNA was isolated from the kidney of the three day 65 fetuses. The genomic DNA was divided into five groups and treated as follows: 1) untreated DNA, 2) DNA treated with the CpG methyltransferase M. Sss1 (methylates all CpGs), 3) DNA treated with M. Sss1 prior to digestion with HpaII, 4) DNA digested with HpaII, and 5) DNA treated with MspI. All groups were amplified by PCR. The primer pair used (Table 2) amplifies a 1108 bp region encompassing exon one through intron two which contains 19 HpaII/MspI sites.

## Results

### Baseline imprinted gene expression in BWS-associated genes in bovids

In order to determine if bovids could be used as a model to study BWS we must first determine baseline expression of imprinted genes known to be misregulated with BWS. Three *B. t. indicus x B. t. taurus* F1 concepti were collected on day 65 of gestation (Figure 1). The brain, tongue, heart, liver, and chorioallantois were analyzed for imprinted gene expression of *KCNQ1OT1*, *CDKN1C*, *PLAGL1*, and *H19*. In cattle, *KCNQ1OT1*, *CDKN1C*, and *H19* are located on chromosome 29 while *PLAGL1* is found on chromosome 9.

**Figure 2:**
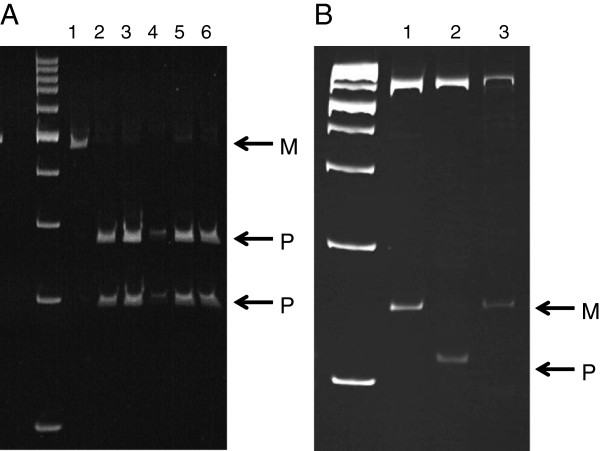
**Allele-specific expression of *B. t. indicus *x *B. t. taurus *F1 concepti.** Shown are two examples of the RFLP assay used to distinguish parent-specific gene expression in *B. t. indicus* x *B. t. taurus* F1concepti. DNA sequence polymorphisms between *B. t. indicus* and *B. t. taurus* were used as a diagnostic test to identify the parental allele origin of the transcript. **A.***KCNQ1OT1* (paternally-expressed gene). Lanes = 1: *B. t. taurus* liver; 2: *B. t. indicus* kidney, 3: *B. t. indicus* fat; 4: F1B heart, 5: F1B liver; 6: F1C heart. **B.***H19* (maternally-expressed gene). Lanes = 1: *B. t. taurus* muscle; 2: *B. t. indicus* fat; 3: F1A heart. M = maternal allele, P = paternal allele.

RFLP was the method used to determine allele-specific imprinted gene expression using SNPs identified by our lab (Figure 2). *KCNQ1OT1*, *CDKN1C*, *PLAGL1*, and *H19* showed the correct monoallelic expression in all tissues analyzed (Table 4). Nonetheless, gene expression was not detected in every tissue of each F1 conceptus studied (Table 4). For example, the RNA of the chorioallantois that belonged to *B. t. indicus x B. t. taurus* F1-C appeared to be degraded because no detectable expression was observed for any RNA assay.

**Table 4:** **BWS-associated imprinted gene expression in *B. t. indicus *x *B. t. taurus *F1**

**Tissue**	**KCNQ1OT1**	**H19**	**CDKN1C**	**PLAGL1**
Conceptus A	(%)- expression from repressed allele			
Chorioallantois	Mono (2.65%)	Mono (0%)	Mono (0%)	Mono (3.75%)
Liver	Mono (6.90%)	Mono (0%)	Mono (0%)	Mono (4.73%)
Brain	Mono (6.01%)	Mono (0%)	Mono (0%)	Mono (1.66%)
Heart	N/A	Mono (0%)	Mono (0%)	Mono (2.17%)
Tongue	Mono (4.09%)	Mono (0%)	Mono (0%)	Mono (6.39%)
Conceptus B				
Chorioallantois	Mono (2.33%)	Mono (0%)	Mono (0%)	Mono (5.50%)
Liver	Mono (6.17%)	Mono (0%)	Mono (0%)	N/A
Brain	Mono 6.46%	Mono (0%)	Mono (0%)	Mono (2.63%)
Heart	Mono (8.01%)	Mono (0%)	Mono (0%)	Mono (4.59%)
Tongue	Mono (7.08%)	Mono (0%)	Mono (0%)	Mono (9.58%)
Conceptus C				
Chorioallantois	N/A	N/A	N/A	N/A
Liver	Mono (4.01%)	Mono (0%)	Mono (0%)	Mono (2.40%)
Brain	Mono (5.60%)	Mono (0%)	Mono (0%)	Mono (4.77%)
Heart	Mono (9.74%)	Mono (0%)	Mono (0%)	Mono (5.74%)
Tongue	Mono (1.96%)	Mono (0%)	Mono (0%)	Mono (0%)

Imprinted Gene expression analyzed by RFLP. Mono= monoallelic expression.

Several of the tissues studied had some level expression from the repressed allele of *KCNQ1OT1*, *CDKN1C*, *PLAGL1*, however because this expression was not greater than 10% they were considered to be expressing those genes in a monoallelic manner. We amplified and digested *B. t. taurus* and *B. t. indicus* to serve as controls for restriction enzyme digestion patterns and to differentiate between leaky expression of the repressed alleles and incomplete restriction enzyme digestion of the tissues. Repression of the paternally-inherited allele of *H19* appeared complete.

### Baseline methylation in BWS-associated imprinting control regions in bovids

**Figure 3:**
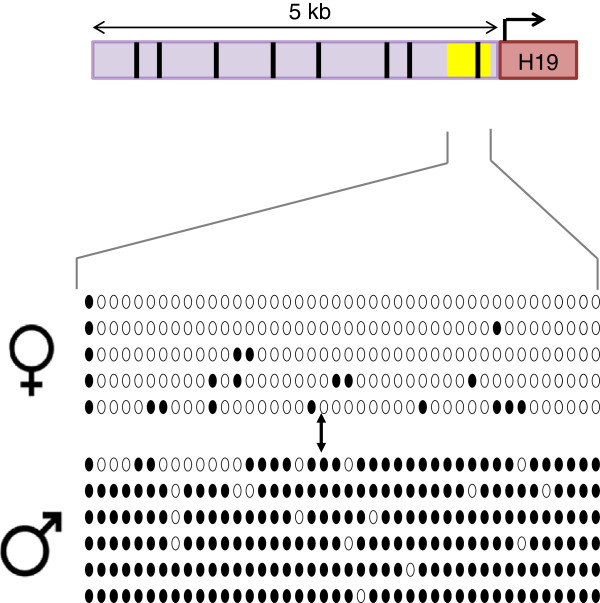
**Differential methylation at the *H19/IGF2 *ICR in bovine.** Top. The putative *H19/IGF2* ICR is drawn to scale and depicted in light purple. Arrow mark the start and direction of *H19*′s transcription. The region amplified by the bisulfite specific primers is represented by a yellow box and encompasses a putative CTCF site. Putative CTCF sites were determined using the University of Essex CTCF searching database (http://www.essex.ac.uk/bs/molonc/binfo/ctcfbind.htm) and are depicted by black vertical lines. From left to right CTCF site 1(cgttaagggg – located at −4739 to −4749 bp from H19′s transcription start site). CTCF2 (ccgcgaggcggcag −4311 to −4325 bp), CTCF3 (ccgcggggcggcgg −3882 to −3896 bp), CTCF4 (cgttaagggg −3372 to −3382 bp), CTCF5 (ccgcgaggcggcag −2944 to −2958 bp), CTCF6 (tggacagggg −1739 to −1749 bp), CTCF7 (ccgcgaggcggcgg −1492 to −1506 bp), CTCF8 (tgttgagggg −251 to −261 bp). Bottom. Shown is an example of bisulfite sequence data from an F1 individual. The bisulfite converted DNA was amplified and cloned prior to sequencing. Each line of circles represents individual alleles. Open circles represent unmethylated CpGs and closed circles represent methylated CpGs. Female symbol = maternal alleles, male symbol = paternal alleles. The position of the SNP used to differentiate between *B. t. indicus* and *B. t. taurus* alleles is shown by an arrow.

**Figure 4:**
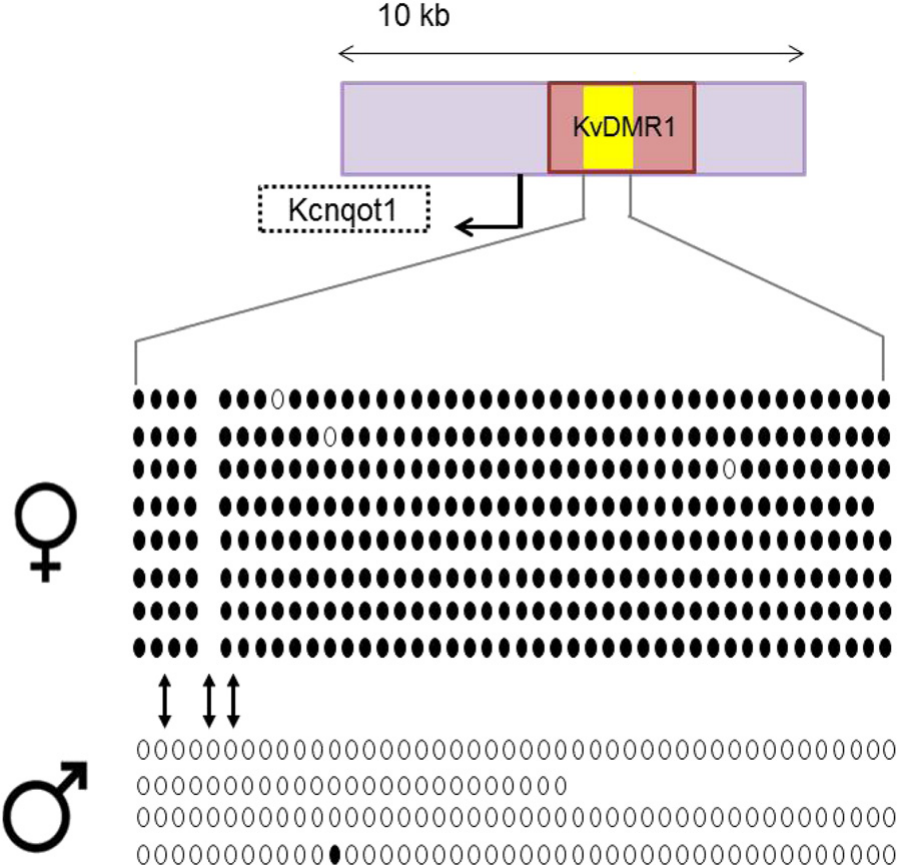
**Differential methylation at the KvDMR1 in bovids.** Top. Part of KCNQ1 10^th^ intron is drawn to scale and depicted in light purple. Arrow depicts direction of *KCNQ1OT1’s* transcription. The region amplified by the bisulfite specific primers is represented by a yellow box. Bottom. Shown is an example of bisulfite sequence data from an F1 individual. The bisulfite converted DNA was amplified and cloned prior to sequencing. Each line of circles represents individual alleles. Open circles represent unmethylated CpGs and closed circles represent methylated CpGs. Female symbol = maternal alleles, male symbol = paternal alleles. The position of the SNP used to differentiate between B. t. indicus and B. t. taurus alleles is shown by arrows. The insertion/deletion “GCG” SNP (Table 2) results in an additional CpG site on the paternal alleles compared to maternal alleles.

COBRA (data not shown) and Bisulfite sequencing were used to determine the methylation status of the *H19/IGF2* ICR (Figure 3) and the KvDMR1 (Figure 4). These two ICRs are the two differentially methylated regions primarily misregulated in BWS patients [9]. From our study we were able to determine that differential methylation is observed within these ICRs in control *B. t. indicus x B. t. taurus* F1 concepti. Both the KvDMR1 and the *H19/IGF2* regions in the bovine showed differential methylation between the parental alleles similar to what has been observed in humans [47-50].

### Methylation analysis of *CDKN1C*’s putative DMR in bovids

**Figure 5:**
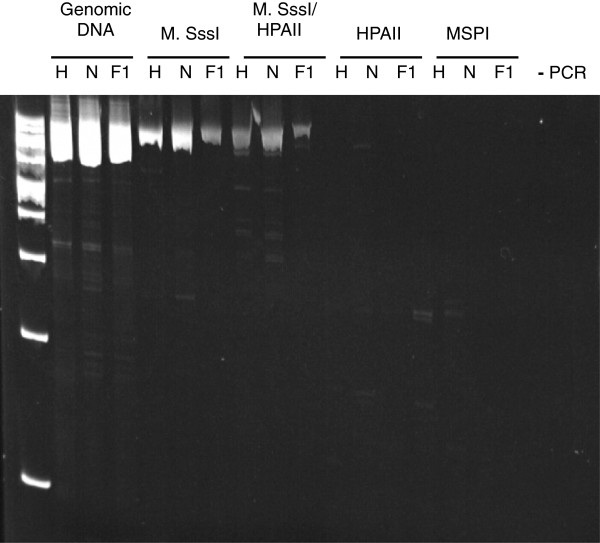
**Methylation analysis of *CDKN1C’s *DMR in bovine.** Restriction enzyme analysis was used to determine the methylation status of *CDKN1C* DMR in the bovine. The restriction enzymes HPAII (blocked by CpG methylation) and MSPI (able to digest both methylated and unmethylated CpGs) were used to determine the methylation of *CDKN1C* exons 1 through intron 2. M. Sss1 (methylates all CpGs) was used as a positive control to show that HPAII is unable to cleave methylated CpGs. Our results show that at least one of the 19 CCGG recognition sites for HPAII was unmethylated because there was no PCR amplification of this region for the HPAII digested template. H = Holstein, N = Nelore, F1 = *B. t. indicus x B. t. taurus* F1-C conceptus. - PCR = water PCR control to show no DNA contamination.

The PCR primers were able to amplify a region of the correct size for the untreated genomic DNA, the M. Sss1 treated DNA, and the M. Sss1 + HpaII treated DNA groups. As expected, MspI digestion cleaved the DNA thus fragmenting the template and preventing amplification of the region (Figure 5). No amplicons were detected for the genomic DNA treated with HpaII suggesting at least one hypomethylated CpG in this genomic region.

## Discussion

In this study, we set out to determine the pattern of expression in bovids of four imprinted genes associated with the human overgrowth syndrome Beckwith-Wiedemann. We analyzed gene expression and DNA methylation in embryonic and extraembryonic tissues of three day 65 *B. t. indicus* x *B. t. taurus* F1 concepti. By using RT-PCR and RFLP analysis we were able to determine the imprinted gene expression for *KCNQ1OT1*, *PLAGL1*, *CDKN1C*, and *H19*. Our results showed that similar to humans, *KCNQ1OT1* and *PLAGL1* are monoallelically expressed from the paternal allele while *CDKN1C* and *H19* are maternally-expressed genes. The imprinted gene expression was observed in all tissues analyzed which included brain, heart, liver, tongue, and chorioallantois.

Another result from this study confirmed recent observations [40] that the KvDMR1 and the *H19/IGF2* ICRs are differentially methylated in cattle as has been reported for human and mouse. Our results add to the current knowledge because of our ability to unequivocally assign methylation status of these ICRs to each parental allele based on the identified SNPs. Results from this work suggest that the *CDKN1C*’s promoter is hypomethylated in bovine as it is in human. This is in accordance with Hori *et al*. [40] who has recently reported a hypomethylated state of the aforementioned promoter.

The imprinted genes associated with BWS have been shown to be conserved between the human and mouse [51-56]. However, there have been several mouse models which have not been able to recapitulate all the diagnostic clinical features associated with BWS [39,57]. No current animal models are able to fully phenocopy BWS. This fact is important for investigators with the goal treating BWS symptoms.

There are many reasons to propose the use of bovids as a model to study BWS. First, LOS has several phenotypical similarities with BWS [30,31,33,37,38]. Second, increased *IGF2* expression has been observed in day 70 LOS concepti [32]. This is of relevance since 2–10% of BWS patients have biallelic expression of the paternally-expressed *IGF2* in tongue and in fibroblast [58]. In BWS, *IGF2*’s biallelic expression is due to gain of methylation on the paternal allele at the *H19/IGF2* ICR. Third, the parent-specific expression pattern of several imprinted genes in the mouse is not conserved in humans (*i.e*. *Gatm*, *Dcn*, and *IGF2r*; [59-63]). Fourth, comparative genome analyses [64,65] show that the percent identity between the genomes of cattle and human is 73.8% while the percent identity between the mouse and human genomes is 66.8% [66]. In addition, pairwise alignments with the human genome of putative transcriptional regulatory regions show a higher homology for cow than for mouse (~80% vs. ~70% [66]). Fifth, as expected given the genomic similarity between human and bovine, we show here that there is conservation of expression and methylation patterns at the BWS-associated loci. Sixth, both species have a nine month gestation period. This is relevant because the sequence of events that result in a condition may occur at similar times during pregnancy. Seventh, both the bovine and human gestation usually involves one offspring. It is likely that there has been divergence for growth regulation of the conceptus between litter bearing and non-litter bearing species.

Another important similarity between humans and ruminants is the adverse response of preimplantation embryos to *in vitro* manipulations. For instance, children that are conceived by the use of assisted reproductive technologies have a higher incidence (3–9 times) of having the LOI overgrowth syndrome BWS [23,26,48,67-70]. Likewise, a fetal overgrowth syndrome has also been documented in ruminants as a result of ART. In ruminants this syndrome is known as LOS. Since the overgrowth phenotype has been observed in ruminants and humans as a result of assisted reproduction, we [71] and others [40] have proposed that both syndromes have similar epigenetic etiologies. In order to determine the plausibility of our hypothesis we need to ascertain if all BWS-associated imprinted gene expression misregulation is recapitulated in LOS. Ongoing studies from our laboratory are determining if LOS and BWS are epigenetically similar.

## Conclusion

In conclusion, our study established the imprinting status of *KCNQ1OT1*, *CDKN1C*, *PLAGL1*, and *H19* in bovine day 65 *B. t. indicus* x *B. t. taurus* F1concepti and found that imprinting was conserved with humans. These genes are associated with the human overgrowth and loss-of-imprinting syndrome BWS. We have also determined that the ICRs primarily affected in BWS, namely KVDMR1 and *H19/IGF2*, are differentially methylated in bovids as in humans. Currently no animal models are able to fully recapitulate BWS. Our results suggest that bovids may be able to serve as an appropriate animal model for studying BWS.

## Competing interests

The authors declare that they have no competing interests

## Authors’ contributions

KMR – performed the majority of the work presented in this manuscript and drafted the manuscript. ZC – optimized the PCR conditions used to amplify the KvDMR1 and analyzed allele-specific methylation of the KvDMR1. KDW – assisted with genome sequence alignments to identify the imprinted loci in the bovine genome and finalized the manuscript. RMR – conceived and designed the project and finalized the manuscript. All authors read and approved the final manuscript.
